# A review on corona virus and treatment approaches with *Allium sativam*

**DOI:** 10.1186/s43094-021-00310-7

**Published:** 2021-08-10

**Authors:** Rupesh Kumar Pandey, Ravindra Kumar Pandey, Shiv Shankar Shukla, Priyanka Pandey

**Affiliations:** 1 Department of Pharmacology KSCP, Swami Vivekanand Subharti University, Meerut, Uttar Pradesh India; 2 Columbia Institutes of Pharmacy, Raipur, Chhattisgarh India; 3 Swami Vivekanand College of Pharmacy, Indore, Madhya Pradesh India

**Keywords:** Ayurveda, Allicin, Antiviral, Lung edema, Angiotensin-converting enzyme, Cytokines

## Abstract

**Background:**

Recently reported cases of Covid-19 globally remind us that new diseases are coming while we are unable to provide the treatment for the same. The entire world is facing this viral attack; deaths are increasing day by day as well as infected patients too. Today, in the period of this disease, can we go to the shelter of our traditional medicines?

**Main body:**

In this article, we have taken medicines related to corona and conceptualized their mechanism, which gave us a chance to understand Garlic's mechanism of action, how Garlic can be a weapon in the lane with this disease. This article also tells how we can treat new diseases with our traditional herbs if no modern medicine has been discovered yet.

**Conclusion:**

The present review is based on the structure of the virus and the targeted site for the drug discovery process with important constituents of *Allium sativam*. The review work also explains the allicin chemical constituent of *Allium sativam* which has targeted therapeutic sites related to Covid-19.

## Background

The pandemic time and concern are Coronavirus which has strains that cause potentially deadly diseases in mammals and birds. The spread of disease in humans is through airborne droplets of fluid produced by infected individuals, some rare but distinguished strains, including 2019-Ncov and those responsible for the severe acute respiratory syndrome. The inception of the disease started in Wuhan China and spread to the whole world [1]. China faced a vulnerable situation in 2004 too, some points which have to be taken seriously that might be the reason for these diseases are air quality index, increased population, and many more which is relevant to the climate.

These viruses are zoonotic, which means they can be spread between animals and people. Available Data suggest the spread of the virus SARS-CoV from civet cats to humans and MERS-CoV from dromedary camels to humans. It's to be noted that we still have various known coronaviruses which are circulating in animals that have not yet infected humans [2]. The Ayurveda has proven efficacy so many times. The recent work is based on efficacious *Allium sativum* which can give a direction towards treatment approaches.

### Common signs of infection

Respiratory symptoms fever and cough shortness of breath and breathing difficulties. The severity of the disease may be attributed to pneumonia, severe acute respiratory syndrome, kidney failure, and even death [2].

## Main text

### Virus structure

The Coronaviruses belong to the family *Coronaviridae* in the order *Nidovirales* [3]. They can be further classified into four genera: *Alphacoronavirus*, *Betacoronavirus*, *Gammacoronavirus*, and *Deltacorona virus*. The alpha- and beta coronaviruses infect mammals, where gammacoronaviruses infect avian species, and delta coronaviruses infect both mammalian and avian species [4].

The structural studies suggest that Coronaviruses are large, enveloped, positive-stranded RNA viruses and have the largest genome among all RNA viruses, typically ranging from 27 to 32 kb. Inside a helical capsid, genome is packed formed by the nucleocapsid protein (N) and further surrounded by an envelope that is associated with at least three structural proteins. The structural studies also implicate the presence of various membrane proteins i.e. membrane protein (M) and the envelope protein (E) are involved in virus assemblage, where the spike protein (S) mediates plays an important role in virus entry in host cells [5].

#### Hemagglutinin esterase

Is a glycoprotein that helps in viruses for invading mechanisms. Several coronaviruses also have envelope-associated hemagglutinin-esterase protein (HE). These help in the attachment and destruction of certain sialic acid receptors that are found on the host cell surface [6].

#### Spike protein

Coronavirus contains spike protein which has a multifunctional molecular machine that helps in coronavirus entry into host cells. Based on Structural study, it has been revealed that a mechanism is based on binding to the receptor on the host cell surface through its S1 subunit and then through the S2 subunit it fuses viral and host membranes [5].

They exist in two structurally distinct conformations, pre-fusion, and post-fusion. Membrane fusion requires the development from pre-fusion to post-fusion conformation in the spike protein. The ACE2 binding triggers a conformational change in the SARS-CoV spike, which exposes previously cryptic Protease sites for cleavage. The role of ACE2 binding in triggering membrane fusion Waits to be further investigated however, SARS-CoV entry does not depend on low pH and at least two protease cleavages in the spike through lysosomal proteases, extracellular proteases, or cell surface proteases [5].

**Fig. 1:**
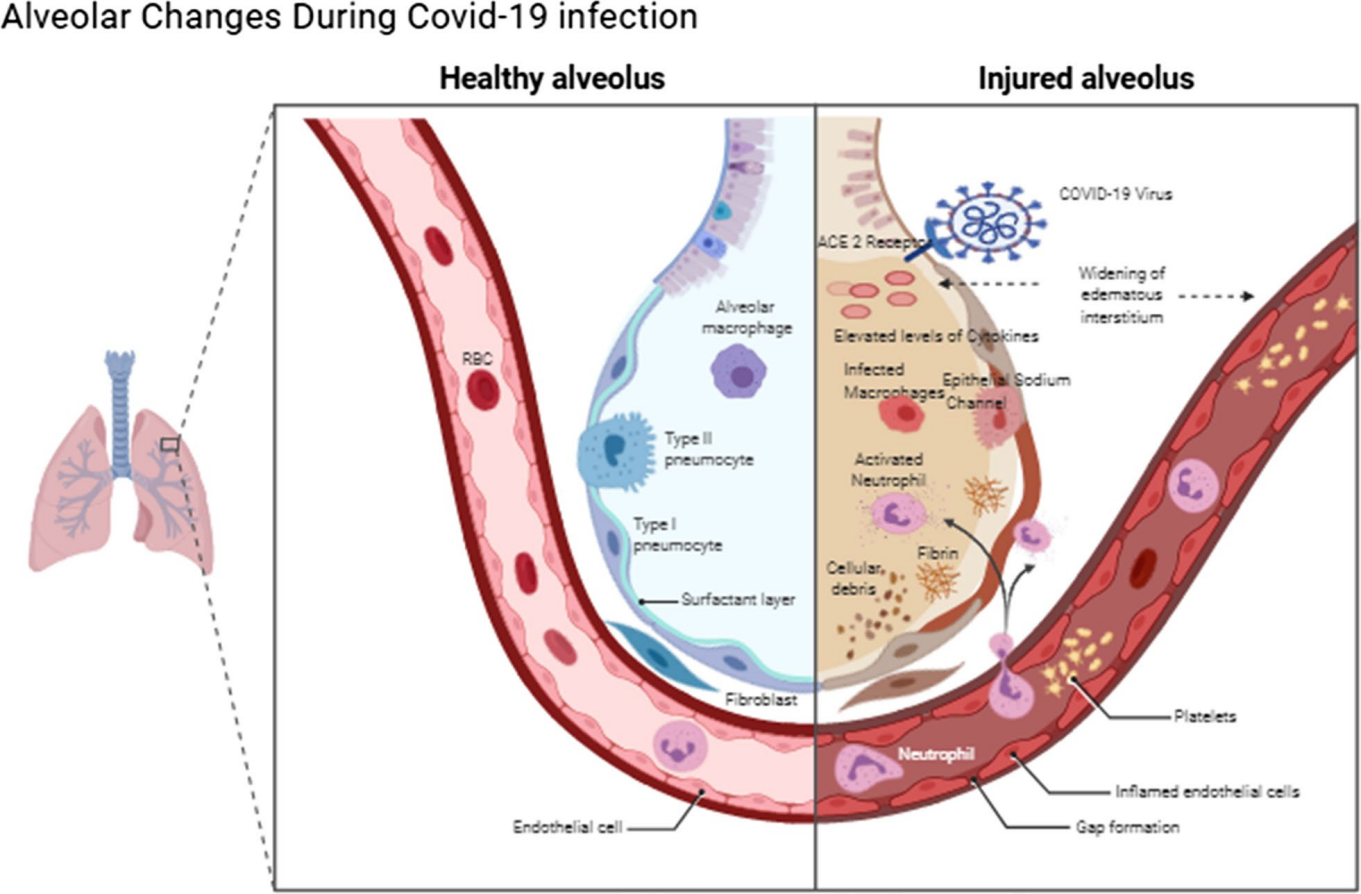
Alveolar changes during covid-19 infection

In general cell entry mechanism of MERS-CoV is similar to that of SARS-CoV. Like the SARS-CoV spike, the MERS-CoV spike must be cleaved at both the S1/S2 boundary and the S2 site for membrane fusion to occur (Fig. 1).

## Allium sativam

*Allium sativum*, Family: Amaryllidaceae is a time-tested herb that is used very commonly in India as a spice. In the traditional medicine system, the herb has proven efficacy in many diseases [7].

The *Allium sativum* contains many constituents like allicin, allyl methyl thiosulfinate, ajoene, and methyl allyl thiosulfinate.

## Proposed therapeutic efficacy of *Allium sativum* in COVID-19 patients

The proposed efficacy is based on reported work up till now with correlation to disease progression. We aim to highlight the efficacy of *Allium sativum against the* COVID-19 through available scientific data.

### Antiviral action and effect on ACE

Thiol enzymes have action on cysteine level which is less in COVID-19 Patients. The major chemical constituent of *Allium sativum,* Allicin acts by preventing several thiol enzymes; other constituents like ajone's have proven their efficacy in viral diseases through leukocytes prevention mechanism [8].

Some researchers revealed that the preventive action of *Allium sativum* against various viruses like influenza B, human rhinovirus type 2, human cytomegalovirus (HCMV), Parainfluenza virus type 3, herpes simplex type 1 and 2, vaccinia virus, and vesicular stomatitis virus [9].

Chemical constituents isolated from *Allium sativum* can inhibit adhesive interaction and fusion of leukocytes which leads by enhancement of natural killer cell (NK cell) activity which destroys the infected virus cells [10].

Many reviews suggested that allicin and S-allylcystein were found in *Allium sativum* inhibit the ACE receptor through the production of Hydrogen Sulfide (H2S) and stimulation of Nitric Oxide (NO), with blockage of α adrenergic receptors and calcium channels [11]. Some studies suggested that extracts of *Allium sativum* can prevent from influenza A (H1N1) virus by inhibiting the nucleoprotein synthesis of virus and polymerase Activity [12].

### Reduction of lung edema through inhibition of epithelial sodium channel

The epithelial sodium channel (ENaC) is an essential sodium selective ion channel that has three subunits, alpha, beta, and gamma. ENaCs expressed the rate-limiting step for the transepithelial absorption of sodium. The transport of sodium through the transepithelial generates osmotic gradients across epithelia, which as a result forces the osmotic transepithelial movement of water; Therefore ENaCs are key factors in the regulation of salt and water homeostasis in various organs [13].

The finding of ENaCs in the lungs are expressed in the airway epithelia which regulates the volume and composition of airway lining fluid and helps alveolar fluid clearance in the alveolar epithelium. In the lungs, increased ENaC activity in the airways can promote cystic fibrosis-like lung disease, whereas ENaC hypoactivity in the distal lung is associated with the formation of pulmonary edema [14].

The garlic compound allicin inhibits ENaC Characteristic through organosulfur compounds S-allyl-Lcysteine, alliin, allicin, and diallyl sulfides. Alliin is situated in the cytosol of garlic cells and which converted into allicin by the enzyme allinase. However, allinase is located in the vacuoles of the cells [15, 16].

### Reduction of pro-inflammatory cytokines and chemokines

The pro-inflammatory cytokines and chemokines are produced by activated macrophages and they are said to be active on inflammation predominantly. In COVID-19 patients the major concern is hyperproduction of pro-inflammatory cytokines and chemokines which leads the disease progression [17].

Some researchers reported that allicin can decrease the level of pro-inflammatory cytokines on systemic and tissue levels. Allicin also modulates the production of IL-1ß, IL-6, and TNF-alpha at mRNA and different protein levels reported in in-vitro studies [18, 19, and 20].

### Action on reactive oxygen species (ROS), inflammatory macrophage infiltration

The free radical scavenging is an essential function that takes care of our body through homeostasis. In a disease state, reactive species and macrophages play a vital role; the chemical constituents like allyl methyl sulfide and diallyl sulfide play a key role by inhibiting angiotensin-II-stimulated cell-cycle sequence, migration, and generation of reactive oxygen species (ROS) that indicates its efficacy in hypertension [21].

Allicin has the ability to break the lipid-soluble chain, which clearly explains its natural antioxidant property, concentration in the brain through crossing the blood–brain barrier, and accumulate at therapeutic levels in the brain. Allicin proved its efficacy by preventing reactive oxygen species damage by up-regulating enzymes that are involved in phase II detoxifying and by accelerating the cellular glutathione level [22].

Allicin may be efficacious due to its modulating property through the enzymatic activity of SH-containing enzymes by a thiol-disulfide exchange reaction. Some studies suggest that allicin has SH-modifying properties that show biological activity by inhibiting the LDL degradation which in turn shows its affinity to free thiol groups and blocks the LDL to macrophage receptors [23].

Allicin can be a good candidate drug for atherosclerosis and reduce plasma lipid concentrations and low-density lipoprotein receptor (LDLR), as it show the efficacy through modification and inhibition of LDL uptake, degradation by macrophages [24, 25].

### Protects mitochondrial function

The mitochondrial functions are essential for the cells for energy-related functions. In COVID-19 Patients the mitochondrial damage causes platelet damage and apoptosis [26]. Allicin inactivates the mitochondrial cytochrome, which is a major factor that activates multiple downstream signaling pathways in ischemic conditions to execute cell death [27].

### In silico studies of allicin against coronavirus

Recent research based on in silico non-covalent and covalent docking screening methods implicates that allicin shows dual *S*-thioallylation of Cys-145 and solvent-exposed Cys-85/Cys-156 residue of SARS-CoV-2 Mpro which acts as a potent inhibitor of SARS-CoV-2 Mpro [28].

## Conclusions

It is understandable from some key points of this paper that we can consider Allicin as a targeted drug for this corona pandemic if we explore the activity of Allicin and Garlic more, then perhaps we can be found the correct directions in the research of COVID'S new medicine.

The effect on ACE and other effects correlate with the efficacy of the drug in treatment, but it's early to say. More research work is to be needed to strengthen the suggested action. We will try to emphasize these points in future research work.

## Data Availability

All data and material are available upon request.

## Abbreviations

Ncov: Novel corona virus
SARS-CoV: Severe acute respiratory syndrome coronavirus
MERS-CoV: Middle East respiratory syndrome related to coronavirus
ACE2: Angiotensin converting enzyme 2
LDL: Low density lipoproteins
